# Dynamic Microtubule Arrays in Leukocytes and Their Role in Cell Migration and Immune Synapse Formation

**DOI:** 10.3389/fcell.2021.635511

**Published:** 2021-02-09

**Authors:** Aglaja Kopf, Eva Kiermaier

**Affiliations:** ^1^CeMM Research Center for Molecular Medicine of the Austrian Academy of Sciences, Vienna, Austria; ^2^Department of Dermatology, Medical University of Vienna, Vienna, Austria; ^3^Ludwig Boltzmann Institute for Rare and Undiagnosed Diseases, Vienna, Austria; ^4^Life and Medical Sciences Institute, Immune and Tumor Biology, University of Bonn, Bonn, Germany

**Keywords:** microtubules, leukocytes, cell migration, immune synapse, cell coherence

## Abstract

The organization of microtubule arrays in immune cells is critically important for a properly operating immune system. Leukocytes are white blood cells of hematopoietic origin, which exert effector functions of innate and adaptive immune responses. During these processes the microtubule cytoskeleton plays a crucial role for establishing cell polarization and directed migration, targeted secretion of vesicles for T cell activation and cellular cytotoxicity as well as the maintenance of cell integrity. Considering this large spectrum of distinct effector functions, leukocytes require flexible microtubule arrays, which timely and spatially reorganize allowing the cells to accommodate their specific tasks. In contrast to other specialized cell types, which typically nucleate microtubule filaments from non-centrosomal microtubule organizing centers (MTOCs), leukocytes mainly utilize centrosomes for sites of microtubule nucleation. Yet, MTOC localization as well as microtubule organization and dynamics are highly plastic in leukocytes thus allowing the cells to adapt to different environmental constraints. Here we summarize our current knowledge on microtubule organization and dynamics during immune processes and how these microtubule arrays affect immune cell effector functions. We particularly highlight emerging concepts of microtubule involvement during maintenance of cell shape and physical coherence.

## Introduction

The cytoskeleton plays a major role for accomplishing numerous immune cell effector functions. Microtubule filaments are crucially important for directing migratory cells to their final destination and for mediating specific cell-cell interactions between an antigen-presenting cell and a T cell. Microtubules are composed of α-/β-tubulin heterodimers which polymerize in a head-to-tail fashion to form a polar protofilament with the α-tubulin subunit exposed to one end – designated as minus-end – and β-tubulin exposed to the other end, which is commonly referred to as the plus-end (Nogales, 2000). In most eukaryotic cells, 13 protofilaments associate laterally to form long, hollow tubes, which are highly dynamic and stochastically oscillate between periods of growth and shrinkage, a property known as “dynamic instability” (Mitchison and Kirschner, 1984). Within cells, microtubule minus-ends are mainly static and stably anchored to a variety of microtubule-organizing structures, from which individual filaments nucleate with their growing plus-ends projecting to the cell periphery (Wu and Akhmanova, 2017). The microtubule lattice as well as the microtubule plus-ends are decorated with microtubule associated proteins (MAPs) such as end-binding (EB) proteins, microtubule (de)-polymerases and regulatory kinesins, all of which modify the dynamics of microtubule filaments and their plus-ends and contribute to highly plastic and functionally specialized microtubule arrays (Bodakuntla et al., 2019). Besides the plethora of MAPs, distinct tubulin isoforms and posttranslational tubulin modifications (PTMs), which collectively make up the tubulin code, introduce additional diversity of microtubule networks (Gadadhar et al., 2017). Today it is well recognized that microtubule structure and dynamics are differentially regulated in a cell-type dependent manner thereby supporting specific morphologies and functions. Here we summarize our current understanding and recent advances of microtubule arrays and their functions in leukocytes.

## Leukocytes

**FIGURE 1:**
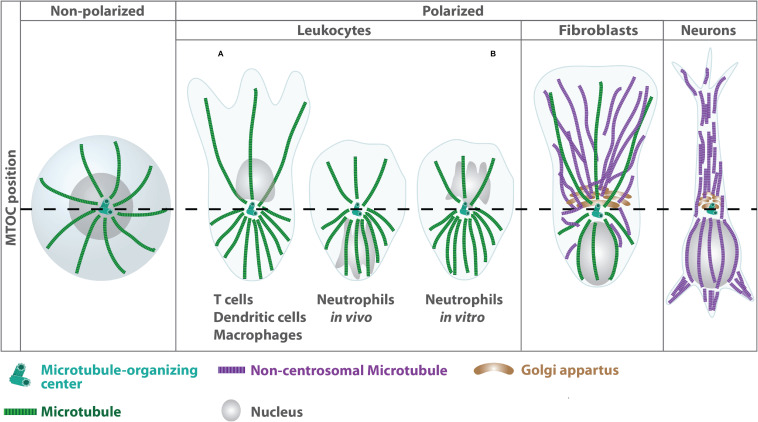
Differential organization of microtubule arrays in distinct cell types. Terminal differentiation into effector cells such as neurons is associated with a reorganization of centrosomal microtubule filaments into non-centrosomal arrays (Muroyama and Lechler, 2017). In highly specialized leukocytes, the centrosome acts as single MTOC, from which a radial array of microtubule filaments project to the cell periphery. In unpolarized leukocytes, the centrosome is located centrally close to the nucleus (left panel). During polarization the MTOC reorients toward the uropod leading to higher densities of microtubule filaments at the cell’s back (right panel). By contrast, fibroblasts exhibit an anterior MTOC localization relative to the nucleus. MTOC positioning is highly plastic in neutrophils and differs depending on the complexity of the environment (Yoo et al., 2013).

Leukocytes are commonly known as white blood cells, which constitute the major component of the body’s defensive unit against diseases. In contrast to erythrocytes, many types of leukocytes exist, which mainly originate from hematopoietic stem cells in the bone marrow. They are classified either by structure into granulocytes and agranulocytes or by cell lineage into myeloid or lymphoid cells. In contrast to other highly specialized cell types, which often reorganize microtubules into non-centrosomal arrays during differentiation, leukocytes primarily nucleate microtubules from the centrally located centrosome with individual filaments extending radially toward the cell periphery (Muroyama and Lechler, 2017; Meiring et al., 2020; Figure 1). One exception to this centrosome-derived microtubule array was described for T cells, in which microtubule nucleation can also proceed from non-centrosomal sites such as the Golgi apparatus (Ong et al., 2018).

One of the most characteristic features of all leukocytes is their highly motile behavior. Lymphocytes constantly patrol the body for foreign antigen by recirculating from blood, through tissue, into lymph and back into the blood stream. Myeloid cells such as neutrophils and monocytes emigrate from the blood stream into tissues when they detect changes on the surface of blood vessels that transmit signals of injury or infection (Springer, 1994). A critical link between innate and adaptive immunity constitute dendritic cells, which, in their resting state, patrol peripheral tissues in search for pathogens (Banchereau et al., 2000). Microbial encounter triggers a maturation process that enables these cells to ingest antigens and become responsive to lymph node homing guidance cues to initiate T cell priming (Steinman and Cohn, 1973). In their mature state, dendritic cells are highly migratory and move through the interstitial matrix to enter the draining lymph node via afferent lymphatic vessels (Worbs et al., 2017). Prior to migration, resting leukocytes respond to specific chemical signals by developing a defined polarized morphology with the formation of an actin-rich lamellipodium at the cell front and a contractile trailing edge at the back, which is referred to as the uropod (Sánchez-Madrid and del Pozo, 1999; Hind et al., 2016). Rearrangement of actin and microtubule filaments are critically involved in establishing this front-rear polarity. Newly synthesized actin polymers at the leading edge protrude the membrane in the forward direction, while myosin-mediated contraction of the trailing edge facilitates de-adhesion and propels the cell body forward. In contrast to stationary polarized cells such as neurons or epithelial cells, leukocytes change their polarity frequently and rapidly, in some cells on a time scale of seconds rather than minutes or hours. Here we focus on the spatial and temporal reorganization of the microtubule cytoskeleton during leukocyte migration in environments of different complexity. We refer the reader to an excellent recent review regarding actin-dependent remodeling of cell locomotion in three dimensional (3D) environments (Yamada and Sixt, 2019).

## Adaptive Migration Strategies Determine the Dependence on Microtubules

Leukocytes exhibit a remarkable repertoire of migratory plasticity, being able to instantaneously switch between adhesion-dependent and adhesion-independent migration. The latter is characterized by the absence of proteolytic degradation and requires frequent cell shape changes in combination with a highly polarized morphology, which bear parallels to the phenotypic behavior of migrating amoeba (Wilkinson, 1986). Due to these morphological similarities, leukocyte motility is commonly designated as being amoeboid, which refers to cell movement driven by frequent shape changes (*amoibe*; Greek for “change”). By contrast, adhesion-dependent migration occurs as a consecutive sequence of leading-edge protrusion, transmembrane force coupling by adhesion receptors and contraction of the cell rear to promote de-adhesion, closely resembling Abercrombie’s three-step mesenchymal migration mode (Abercrombie et al., 1970, 1977).

Adaptive leukocyte migration strategies strongly depend on the environmental context: when moving along sheet like structures such as the endothelial lining of blood vessels or basement membranes, leukocytes exhibit a mesenchymal mode of migration. However, in most cases leukocytes reside in structurally complex microenvironments such as collagen-rich interstitial matrices or cell-rich compartments found in lymph nodes or granulomas. When embedded in such environments, leukocytes shift their mode of migration to an adhesion-independent amoeboid mode (Lämmermann et al., 2008; Renkawitz et al., 2009; Friedl and Wolf, 2010). This migration mode is powered by frequent cell shape changes that intercalate with any textured environment and thus propel a cell forward, thereby enabling leukocytes to move in an autonomous manner, irrespective of the chemical composition of their microenvironment (Reversat et al., 2020). Finally, leukocytes cross the endothelial cell barrier in a process called transendothelial migration (TEM) or diapedesis (Ley et al., 2007; Muller, 2011). While microtubule arrays in endothelial cells play an important role during TEM (Mamdouh et al., 2008), in leukocytes neither preexisting nor *de novo* generated microtubules seem to be essential for efficient transmigration (Fine et al., 2016; Yadav et al., 2019).

**FIGURE 2:**
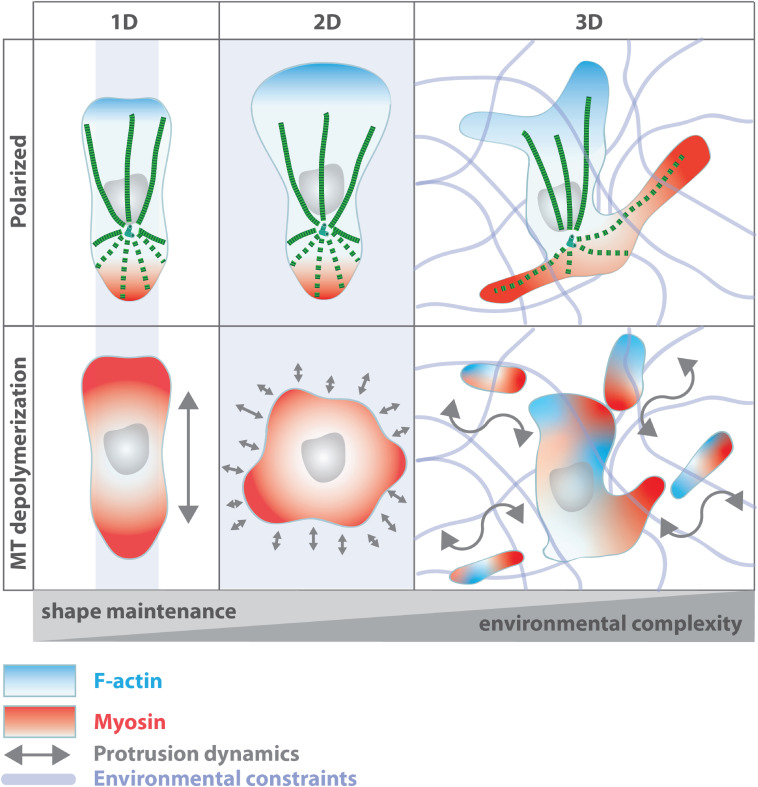
Microtubule function during leukocyte migration in environments of different complexity. Microtubule depletion leads to uncoordinated actomyosin activation, yet with different consequences on cell shape and coherence depending on the geometry of the cell’s surrounding. Oscillating myosin activation across the cortex yields in uncoordinated and unstable polarization in 2D while in linear channels (1D) leukocytes maintain their polarized shape. In complex 3D environments, microtubules mediate the coordination of multiple competing protrusions (Renkawitz et al., 2019; Kopf et al., 2020). Disruption of microtubule integrity impairs protrusion-retraction dynamics of competing extensions resulting in loss of cell shape and induction of cell fragmentation.

A unifying feature of both migration strategies is that, in order to fulfill their effector functions, migrating leukocytes need to integrate the entire imposed mechanochemical parameters of the microenvironment to successfully navigate to their destination site. While actin dynamics are essential for locomotion and cell contractility, dynamic microtubules are indispensable for cell shape and the establishment and maintenance of cell polarity. Much of the gained knowledge originates from studies of cells moving on 2D surfaces, but recent evidence suggests a differential role for microtubules during migration in complex 3D environments (Figure 2), where mesenchymal cells begin to depend on an intact microtubule network to move within soft matrices (Unemori and Werb, 1986; Doyle et al., 2009).

### Microtubule Arrays in Simple Environments

The role of microtubules during leukocyte migration was initially assessed in neutrophils. In the absence of a chemotactic cue neutrophils rest or move without any preferred direction. This non-directed mode of locomotion is referred to as random migration. Uniform addition of a chemotactic factor increases random migration – a phenomenon termed stimulated random migration or chemokinesis. If the chemotactic factor is presented as a gradient, migration changes from a stimulated random mode to directional migration. Under these conditions, cells move toward the chemotactic source *en masse*, which is known as chemotaxis (Allan and Wilkinson, 1978; Zigmond et al., 1981). Various compounds have been identified to bind to tubulin and/or microtubules thereby altering microtubule polymerization and dynamics. Most of these agents originate from natural sources and were isolated from marine organisms, plants and bacteria (Amador et al., 2003). At high concentrations these compounds are often classified into either microtubule-stabilizing agents such as taxol and the epothilones or destabilizing agents, which include colchicine, vinblastine or nocodazole. Most microtubule-stabilizing agents bind to β-tubulin on the luminal side of microtubules and stabilize longitudinal and/or lateral tubulin contacts (Neuert et al., 2013; Alushin et al., 2014). Microtubule-destabilizing agents bind between the α- and β-tubulin subunit within the same dimer or between two different longitudinally aligned dimers. They act on microtubules by different mechanisms all of which de-stabilize the dynamic plus-ends of microtubules (Skoufias and Wilson, 1992; Ravelli et al., 2004; Gigant et al., 2005).

Probing neutrophils with microtubule destabilizing agents impairs direction-finding or directional movement during chemotaxis but not the mechanism of neutrophil locomotion *per se* (Bandmann et al., 1974; Gallin and Rosenthal, 1974; Keller et al., 1984; Niggli, 2003; Xu et al., 2005). A similar phenomenon of diminished persistent locomotion toward chemotactic cues upon disturbing microtubule integrity was also observed in macrophages, T cells and dendritic cells *in vitro* and *in vivo* (Ratner et al., 1997; Redd et al., 2006; Takesono et al., 2010; Yoo et al., 2013; Kopf et al., 2020). Stabilization of microtubules by taxol similarly disturbed the polarized distribution of F-actin and greatly reduced directional locomotion without affecting migration velocities of migrating T cells and neutrophils (Niggli, 2003; Yadav et al., 2019) indicating that dynamic microtubules do not contribute to the force-generating mechanisms required for amoeboid migration but rather support the path of locomotion along the chemotactic gradient. Interestingly, in the absence of chemoattractant, microtubule depolymerization induces neutrophils to spontaneously polarize and migrate randomly suggesting that in resting neutrophils, microtubules rather suppress polarity instead of inducing it (Bandmann et al., 1974; Gallin and Rosenthal, 1974; Dziezanowski et al., 1980; Keller et al., 1984; Niggli, 2003; Xu et al., 2005).

### How Do Microtubules Regulate Directional Locomotion in Simple Environments?

Leukocyte microtubules rapidly respond to chemotactic cues with increased filament polymerization (Gallin and Rosenthal, 1974; Robinson and Vandre, 1995). During cell polarization, the microtubule array reorients toward the uropod, which is maintained during locomotion and does not require microtubule disassembly or substrate attachment through integrin receptors (Malech et al., 1977; Anderson et al., 1982; Ratner et al., 1997; Eddy et al., 2002; Xu et al., 2005). Inhibition of myosin phosphorylation or actin polymerization causes an expansion of the microtubule array and penetration of microtubules into the lamellipodium indicating that F-actin- and myosin II-dependent forces lead to the development and maintenance of microtubule asymmetry (Eddy et al., 2002). During chemotaxis, the microtubule organizing center (MTOC) relocalizes behind the nucleus and microtubules align along the axis of migration with individual filaments orienting toward the uropod while the F-actin rich lamellipodium becomes populated with only few filaments extending to the leading edge cell membrane. One exception to the posterior localization of the MTOC is observed in neutrophils, which change MTOC orientation rapidly depending on the environmental conditions, yet, with individual filaments projecting to the back of the cell (Chiplonkar et al., 1992; Yoo et al., 2013). Relocalization of the microtubule array to the uropod increases microtubule density at the cells’ back suggesting that microtubules promote back-directed processes rather than stabilization of the lamellipodium as reported for mesenchymal migration (Waterman-Storer et al., 1999). This is further supported by the fact that the MTOC follows the turning leading edge instead of guiding the direction of the lamellipodium indicating that the MTOC and microtubule filaments do not determine the cell’s leading protrusion but rather act as a path finder by stabilizing a chosen direction of locomotion (Ueda et al., 1997; Xu et al., 2005).

### How do Microtubules Stabilize the Path of Locomotion?

Recent studies in dendritic cells revealed that the dynamics of individual microtubule filaments are differentially regulated depending on their localization: while microtubules projecting to the leading edge are stable and long lived, microtubule dynamics at the trailing edge exhibit higher frequencies of shrinkage events compared to front-directed filaments. The high local microtubule depolymerization rate at the uropod is causally connected to retraction of the cell’s back demonstrating that microtubules are able to regulate local cellular retraction events (Kopf et al., 2020). Two signaling modules have been identified, which act in concert to balance morphological frontness and backness in migrating cells: RhoA- and actomyosin dependent contractility stimulate cellular backness, while F actin-rich protrusions at the cell front are regulated by a trimeric G protein, the small GTPase Rac, and 3′ phosphoinositides such as PIP3 (Niggli, 2003; Xu et al., 2003; Yoo et al., 2010). Microtubules link RhoA and actomyosin activation by release of the RhoA-specific guanine nucleotide exchange factor (GEF)-H1 (Ren et al., 1998; Krendel et al., 2002; Chang et al., 2008). Microtubule depolymerization leads to untethering of GEF-H1 from the microtubule lattice, which in turn induces RhoA activation and downstream Rho-associated protein kinase (ROCK) signaling leading to myosin II phosphorylation and actomyosin contraction (Krendel et al., 2002; Nalbant et al., 2009). In migrating dendritic cells, microtubules projecting to the back of the cell stabilize the uropod by delivering GEF-H1, allowing for local activation of RhoA and myosin contraction (Kopf et al., 2020). Such subcellular microtubule-mediated activation processes, specifically lead to local contraction events via the RhoA-ROCK-myosin axis as demonstrated for the uropod. This local RhoA activation might explain the different behavior of neutrophils after microtubule depolymerization in the absence of chemotactic cues: while large and ramified dendritic cells allow local microtubule-mediated cell retraction events, in small cells such as neutrophils, microtubule depolymerization globally activates RhoA leading to oscillating cortical myosin activation and fluctuating cell polarization, which in turn induces random motility.

### Microtubules in Complex Environments – Implications for Cell Coherence

The involvement of microtubules, specifically during leukocyte migration in complex environments, still remains an underexplored area of research. On top of that, not only environmental constraints but also cell intrinsic factors such as cell size and protrusion dynamics affect the level to which microtubules are required for migrating leukocytes.

To efficiently navigate through dense 3D microenvironments, leukocytes initially extend multiple protrusions into pores of their immediate vicinity to explore different potential paths for locomotion. Ultimately, they have to retract all but one protrusion thereby selecting one direction along which the cell further advances. The most critical part during migration in complex environments is translocation of the cell’s nucleus, which constitutes the largest organelle within the cell. During path selection leukocytes position their nucleus to the cell front to act as a mechanical ruler allowing to detect pore size differences. Ultimately, cells bypass local hindrances within the extracellular matrix by choosing larger pore sizes thus favoring the path of least resistance (Renkawitz et al., 2019). The MTOC is positioned directly behind the nucleus and seems to push the nucleus forward (Zhao et al., 2012). Once the MTOC passes the junction point, competing cellular protrusions become instable and retract in a microtubule dependent manner (Kopf et al., 2020). Due to their stiff nature, microtubule filaments grow in straight trajectories and fail to enter curved or buckled protrusions. Once protrusions bend beyond a critical point, microtubules start to depolymerize and – via release of GEF-H1 – locally activate RhoA and actomyosin contraction thereby inducing cellular retraction (Figure 2). This constitutes a remarkably simple mechanism of how multiple competing protrusions of ramified cells communicate with each other and establishes the microtubule cytoskeleton as an important mediator of cell integrity. Such subcellular microtubule-mediated actomyosin activation processes, which specifically lead to local retraction events of remote cell edges, seem to work primarily in large and ramified cells, while small cells such as neutrophils and T cells typically employ plasma membrane tension to communicate between multiple competing protrusions (Diz-Muñoz et al., 2016). In contrast to cells moving in geometrically simple environments, global microtubule depolymerization of cells embedded in complex 3D environments causes loss of cell coherence by uncoordinated protrusion dynamics, which ultimately leads to auto-fragmentation and cell death (Renkawitz et al., 2019; Kopf et al., 2020; Figure 2).

Similarly, microtubules also control cell shape in tissue-resident memory T (*T*_*RM*_) cells. Skin *T*_*RM*_ cells exhibit highly dynamic shape changes when migrating within the constrained epidermal layer with extension of multiple competing protrusion similar to dendritic cells. Treatment of *T*_*RM*_ cells in the skin with microtubule destabilization agents induces cell elongation and fragmentation of extending dendrites suggesting that an intact microtubule network is also required to maintain cellular coherence of *T*_*RM*_ cells within the epidermis (Zaid et al., 2017). Besides GEF-H1, the atypical Cdc42-specific GEF dedicator of cytokinesis 8 (DOCK8) plays an important role for maintaining lymphocyte cell integrity. DOCK8-deficient T cells and natural killer cells develop an abnormally elongated shape and deformation of the nucleus in 3D collagen matrices and human skin tissues (Zhang Q. et al., 2014). Chemotaxis of DOCK8-deficient T cells is unaffected but cells fragmented frequently when moving in confined spaces, causing a distinct form of cell death and the inability to generate long-lived tissue resident T cells. These results establish a crucial role for DOCK8 in mediating cytoskeletal rearrangements during locomotion in 3D environments. Whether DOCK8 directly affects microtubule integrity is currently unknown.

In summary, there is mounting evidence that a dynamic microtubule cytoskeleton is essential for maintenance of cell shape and coherence by coordinating protrusion dynamics of leukocytes migrating in complex 3D environments.

### Microtubules and the Formation of the Immune Synapse

The high specificity of immune responses largely depends on direct cell-cell interactions. Leukocytes are able to form tight contacts with other types of immune- or non-immune cells leading to the initiation of adaptive immune responses or activation of effector cells able to eliminate potentially dangerous cells. In analogy to the morphologically similar neuronal synapse, the contact area that is built between two adjacent cells is referred to as the immunological synapse. Two major types of immune synapses exist – helper T cell synapses forming between antigen-presenting cells and T helper cells and cytotoxic synapses, which are established between cytotoxic T cells or natural killer cells and a target cell. Both types of synapses share common features but also display cell type-specific differences. However, in both cases, the effector cells must distinguish foreign antigens from self-proteins, which poses a major challenge to this task in terms of specificity.

### T Helper Cell and Cytotoxic Synapses

Efficient activation of T helper cells is mediated through the presentation of a specific antigen by an antigen-presenting cell. The T cell receptor first recognizes a cognate antigen, which is presented on major histocompatibility complexes on the surface of the antigen-presenting cell (Turley et al., 2000). Subsequently, this interaction leads to the formation of the immune synapse at the T cell-antigen-presenting cell junction. At the immune synapse, T cell receptors, integrins and co-stimulatory receptors engage with each other to form a series of supramolecular activation clusters (SMAC), which segregate into radial symmetric zones facing the antigen-presenting cell (Shaw and Dustin, 1997; Dustin and Choudhuri, 2016). Structurally, the T cell receptors and associated kinases cluster in the central area (central supramolecular activation cluster; central SMAC), while adhesion receptors and actin, as well as actin-interacting proteins, reorganize in surrounding external rings referred to as the peripheral and distal SMAC (peripheral SMAC and distal SMAC) (Monks et al., 1998; Grakoui et al., 1999; Krummel et al., 2000).

Cytotoxic lymphocytes play a crucial role in mediating innate and adaptive immune responses. Natural killer cells and cytotoxic T lymphocytes provide rapid responses to virally infected cells and tumor formation. They are able to directly eliminate cells by the release of large amounts of secretory lysosomes – termed lytic granules – leading to target cell lysis. Impaired functioning of cytotoxic T lymphocytes may lead to immune evasion of tumors and the emergence of chronic infections. Similar to T cell activation, the first step in cell-mediated cytolysis is binding of the cytotoxic T lymphocyte’s T cell receptor to a foreign antigen presented on the surface of the target cell. Cytotoxic synapses structurally display a similar organization as T helper cell synapses with a ring of adhesion molecules surrounding an inner signaling domain. Secretion occurs into a specialized domain lying next to the central SMAC and within the peripheral SMAC. A secretory cleft, which appears as an indentation in the membrane of the target cell, lies opposite the secretory domain (Stinchcombe et al., 2001).

In both cases, T cell receptor-antigen ligation leads to repositioning of the centrosome from the uropod to the contact side: upon ligation of an antigen-loaded B cell with a T helper lymphocyte, the MTOC inside the T cell reorients toward the cell contact region with the antigen-presenting cell (Kupfer et al., 1986; Kupfer and Singer, 1989). α-/β-tubulin heterodimers polymerize from the MTOC and form a network of microtubules at the T cell-antigen-presenting cell contact side. MTOC positioning toward the contact zone strictly depends on T cell receptor engagement with the cognate antigen and concomitantly drives the movement of other organelles such as the Golgi apparatus toward the immune synapse (Kupfer et al., 1986). The rapid repositioning of the T cell’s MTOC allows microtubule plus-end directed transport of secretory vesicles containing effector molecules and polarized secretion of these molecules in the direction of the bound antigen-presenting cell (Kupfer et al., 1991). Similarly, Geiger and colleagues demonstrated relocalization of the MTOC and perinuclear Golgi apparatus toward the synapse upon cytotoxic T lymphocyte-target cell conjunction (Geiger et al., 1982). Disturbing microtubule integrity with nocodazole causes dispersion of the Golgi apparatus and reversibly inhibits target cell lysis indicating microtubule involvement in the delivery of lytic granules toward the immune synapse (Katz et al., 1982; Kupfer and Dennert, 1984).

Polarization of the microtubule cytoskeleton and directional secretion of soluble factors toward the immune synapse emerged as key events allowing for specific activation of effector cells and destruction of potentially dangerous cells. During the past 25 years, numerous studies shed light on the mechanisms and stimuli triggering MTOC repositioning in T cells. By contrast, much less is known about the antigen-presenting cell side of the immune synapse. Here we highlight the molecular basis of centrosome translocation and polarized vesicle secretion in T cells and antigen-presenting cells and the role of the microtubule cytoskeleton during these processes. We refer to excellent recent reviews, which discuss the signaling modules that regulate the repositioning of centrosomes toward the immune synapse (Martín-Cófreces and Sánchez-Madrid, 2018; Garcia and Ismail, 2020; Tittarelli et al., 2020).

### Microtubule Dynamics at the Immune Synapse

In resting cytotoxic T lymphocytes, microtubules are highly dynamic switching constantly between growth and shrinkage. Microtubules contacting the cell cortex buckle, curl inward and slide laterally along the cell cortex or are prone to depolymerize (Kuhn and Poenie, 2002). Upon T cell receptor ligation, microtubule filaments are anchored to the peripheral SMAC, a ring-shaped structure which surrounds the central SMAC and colocalizes with clusters of the T cell’s major integrin LFA-1. Subsequently to the attachment of microtubules to the cell cortex, the MTOC is recruited vectorially to the contact zone. Some microtubules bent at the contact site suggesting the presence of microtubule motor proteins anchored at the cell cortex, which act on microtubules to pull the MTOC toward the immune synapse (Kuhn and Poenie, 2002). Candidate molecules, which have been demonstrated to connect microtubules to the peripheral SMAC at the immune synapse are the cytoskeletal adaptor proteins IQGAP1 and Cdc42-interacting protein 4 (CIP4). IQGAP1 and actin are cleared away from the synapse via an expanding ring, which in turn exerts tension on the microtubule filaments that are anchored at the peripheral SMAC (Stinchcombe et al., 2006). CIP4 interacts with both, the actin and microtubule cytoskeleton and was proposed as functional link between the peripheral SMAC and MTOC polarization (Stinchcombe et al., 2006; Banerjee et al., 2007). MTOC translocation toward the immune synapse further requires precise regulation of microtubule dynamics. Overexpression of histone deacetylase 6, the enzyme that catalyzes the removal of acetylation at lysine 40 of α-tubulin and thus reduces microtubule stability, results in defective MTOC polarization toward the immune synapse (Serrador et al., 2004). Similarly, destabilization of microtubules by either depletion of formins or the microtubule-associated protein 4 (MAP4) impairs MTOC translocation in T cells (Andrés-Delgado et al., 2012; Bustos-Morán et al., 2017). After MTOC polarization, microtubules actively polymerize at the immune synapse. Altering microtubule plus-end dynamics by depletion of the microtubule catastrophe-inducing protein stathmin or the plus-end-binding protein 1 (EB1) as well as impairing EB1 phosphorylation delays MTOC reorientation and movement of vesicles at the immune synapse. Together, these findings highlight the importance of dynamic microtubules in the establishment of a functional immune synapse and T cell activation (Zyss et al., 2011; Filbert et al., 2012; Martin-Cofreces et al., 2012).

A prominent player to generate force for efficient MTOC relocalisation is the microtubule-based motor protein dynein. Dynein acts in concert with two other central components, dynactin and coiled coil containing activating adaptor proteins, which together with additional regulators form a high-molecular weight complex referred to as the cytoplasmic dynein complex (Reck-Peterson et al., 2018). Dynein is composed of six polypeptide chains, one single dynein heavy chain (DHC), two intermediate- and three light chains (dynein intermediate chain (DIC) and dynein light chain (DLC), respectively) all of which are present as two copies. The DHC contains the motor domain that associates with microtubules via a microtubule-binding domain (Kon et al., 2012; Schmidt et al., 2012). Through its direct interaction with the microtubule cytoskeleton, the dynein-dynactin machinery is able to move a variety of different cargoes toward the minus-end of microtubule filaments (Gill et al., 1991; Schroer and Sheetz, 1991).

In stimulated T helper cells, dynein and dynactin colocalize as rings with the adhesion and degranulation promoting adaptor protein (ADAP) at the peripheral SMAC. Similarly, microtubule filaments project to the immune synapse and associate with ADAP into a ring-like structure (Combs et al., 2006). Loss of ADAP diminishes recruitment of dynein and centrosome relocalization suggesting a causal link between ADAP, cytoplasmic dynein localization and MTOC reorientation toward the immune synapse. Accordingly, interference with dynein-dynactin activity by either overexpression of p50-dynamitin or silencing the DHC prevents MTOC relocalization after T cell receptor engagement supporting the notion that cytoplasmic dynein is functionally involved in centrosome polarization toward the immune synapse (Martin-Cofreces et al., 2008).

**FIGURE 3:**
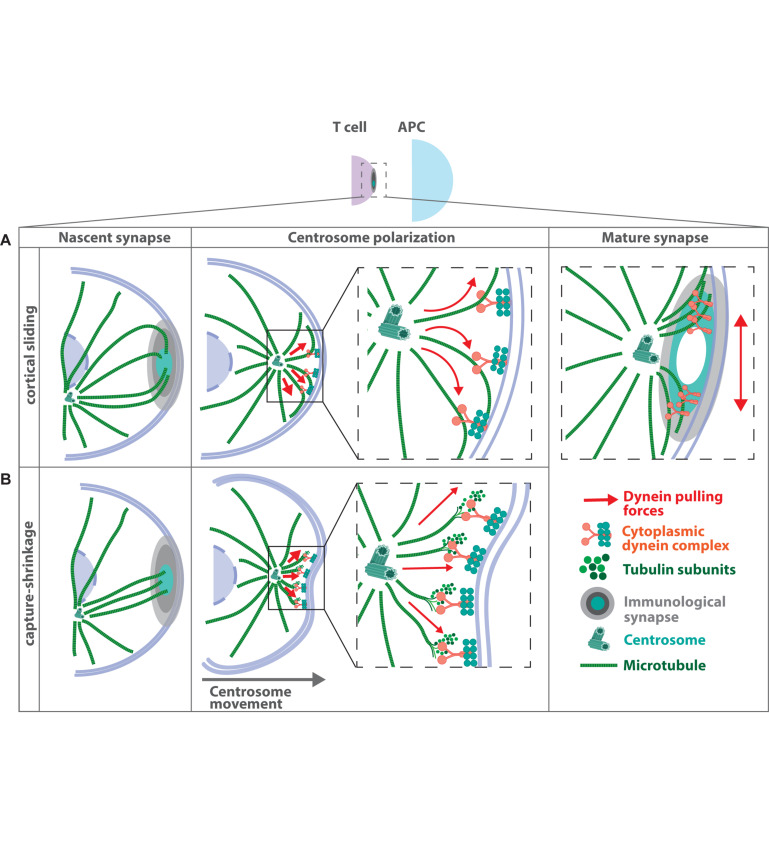
Mechanisms of MTOC translocation during immune synapse formation. Antigen recognition on the surface of an antigen presenting cell by the T cell receptor leads to the formation of a tight contact zone between two adjacent cells. Microtubule filaments inside the T cell project toward the contact area and subsequently the MTOC gets recruited to the immune synapse. Two major models have been proposed how MTOC relocalization is achieved, both of which depend on the presence of the microtubule-associated motor protein dynein at the plasma membrane. **(A)** In the “cortical sliding model” dynein is held in place at the cell cortex and simultaneously walks toward the minus-end of the microtubule filaments. This leads to cortical sliding of microtubules and pulling of the MTOC toward the contact site (Martin-Cofreces et al., 2008). **(B)** The “capture-shrinkage model” suggests that the force, which is required for MTOC translocation is generated by the dynamic instability of microtubule filaments and transmitted via dynein (Yi et al., 2013). Here dynein couples microtubule depolymerization to the cell cortex to move the MTOC toward the synaptic membrane. Recently a revised model for MTOC translocation to the immune synapse was proposed, in which centrally localized dynein first pulls the centrosome straight toward the immune synapse. Afterward, dynein congresses at the peripheral SMAC, which leads to opposing forces acting laterally on the centrosome (Maskalenko et al., 2020). Such lateral pulling forces lead to oscillating movements of the centrosome at the immune synapse.

Provided that microtubule minus-ends are anchored at the MTOC these data support a model, in which the dynein-dynactin complex localizes to the plasma membrane at the immune synapse and exerts the force, which is required to recruit the MTOC close to the contact site (Figure 3A). In this model, dynein is held in place at the cell cortex and simultaneously walks toward the minus-end of the microtubule filaments leading to cortical sliding of microtubules and pulling of the MTOC toward the plasma membrane (Martin-Cofreces et al., 2008). MTOC repositioning based on this cortical sliding mechanism has been assessed by quantitative biomechanical modeling (Kim and Maly, 2009). According to this model, a pulling mechanism is capable of MTOC reorientation and compatible with the vectorial description of MTOC translocation derived from earlier experiments (Kuhn and Poenie, 2002).

A second model based on experimental data suggests that the force, which is required for MTOC translocation is generated by microtubule depolymerization and coupled via dynein to move the MTOC toward the cortex (Figure 3B). In this model, dynein binds to microtubule plus-ends at the plasma membrane and drives MTOC repositioning via a capture-shrinkage mechanism (Yi et al., 2013). Optical tweezers were used to place the antigen-presenting cell to the opposite site of the T cell’s MTOC allowing to image the process of MTOC repositioning and microtubule dynamics in T cells with high spatial and temporal resolution. MTOC repositioning follows two distinct kinetic phases: a fast polarization phase of ∼3.3 μm/min and a slower ∼0.9 μm/min docking phase. Similar to previous studies, microtubule plus-ends project from the MTOC toward the antigen-presenting cell but terminate at the center of the immune synapse in an end-on manner. Several microtubules center at the immune synapse and undergo simultaneous shortening thus bringing the immune synapse and the centrosome together. During microtubule shortening, the membrane underneath the immune synapse invaginates, suggesting force generation at the point of microtubule depolymerization. Similar to the cortical sliding mechanism, inhibition of dynein or microtubule integrity impairs MTOC repositioning (Yi et al., 2013). In support of this, *in vitro* reconstitution experiments showed that barrier-attached dynein is indeed able to trigger microtubule shrinkage and to generate pulling forces, which are sufficient to center microtubule asters in confining geometries (Laan et al., 2012).

Differences in the underlying mechanism of centrosome repositioning most likely arise from the distinct experimental set-ups: T cell-antigen-presenting cell contacts were initially observed using polarization light microscopy in combination with end-stage fixed samples of cell-cell conjugates (Kuhn and Poenie, 2002; Martin-Cofreces et al., 2008). In order to observe the dynamics of centrosome reorientation toward the immune synapse, Yi and coworker established an optical trap, which allows precise control of cell positioning by placing the antigen-presenting cell opposite to the location of the T cell’s centrosome. This allows real-time monitoring and quantitative analysis of MTOC relocalization.

However, in both models dynein anchored at the cell cortex is essential to generate or transmit the force required for MTOC polarization. It is well established that recruitment of dynein and subsequent MTOC translocation depends on the polarized accumulation of the lipid second messenger diacylglycerol (DAG) in the cell membrane (Quann et al., 2009). DAG is generated downstream of T cell receptor ligation by the hydrolysis of phosphatidylinositol-4,5-bisphosphate (PIP2) through Phospholipase C-γ (PLC-γ). Blocking DAG production or disrupting the localization of DAG impairs MTOC polarization in T helper cells and cytotoxic T lymphocytes. Mechanistically, DAG recruits and activates three distinct protein kinase C (PKC) isoforms to the synaptic membrane, which in turn control centrosome reorientation through the localization of dynein and non-muscle myosin II (Quann et al., 2011; Liu et al., 2013). The involvement of non-muscle myosin II suggests that there might be dynein-independent force-generating mechanisms operating to reorient the MTOC. In this regard, pharmacological dynein inhibition in Jurkat T cells, had no effect on MTOC translocation toward the immune synapse (Hashimoto-Tane et al., 2011). Yet, it is hard to estimate whether this might be due to incomplete dynein inhibition or dynein-independent mechanisms of MTOC reorientation. Dynein is further known to form a complex with either Lissencephaly 1 (LIS1) and NudE Neurodevelopment Protein 1 (NDE1), or with dynactin, a multisubunit complex whose largest subunit is p150^Glued^ (Reck-Peterson et al., 2018). Both complexes form mutually exclusive with dynein and mediate distinct cellular activities during T cell activation (Nath et al., 2016). Knockdown of NDE1 or expression of a dominant negative form diminishes dynein localization at the immune synapse and translocation of the MTOC. By contrast, knockdown of p150^Glued^ results in impaired recruitment of secretory vesicles toward the immune synapse whereas MTOC polarization is unaffected. These results suggest that dynein might play a dual role in activating T cells depending on the interacting partner protein (Nath et al., 2016). More recently, a study from the same group demonstrates that the NDE1–dynein complex first accumulates at the center of the immune synapse and then later becomes associated to the peripheral SMAC. The relocalization of dynein depends on the disrupted in schizophrenia 1 (DISC1)-girders of actin filaments (Girdin) complex (Maskalenko et al., 2020). Depletion of either DISC1 or Girdin results in loss of actin accumulation at the immune synapse, impaired recruitment of members of the dynein complex and a failure in MTOC translocation to the synapse. The authors propose a model for MTOC translocation, in which centrally located dynein first pulls the MTOC straight toward the immune synapse until it reaches the center of the dynein ring (Figure 3). When dynein relocates to the peripheral SMAC, opposing dynein forces would act to pull the MTOC laterally in an oscillating manner similar to what has been observed in experimental setups before (Kuhn and Poenie, 2002).

Independently of the mechanism of force generation and transmission, microtubules need to be anchored at the immune synapse to efficiently allow MTOC reorientation. Based on the “search and capture” model, which was first proposed in the context of microtubule-chromosome interaction during mitotic spindle assembly (Hill, 1985; Pavin and Toliæ-Nørrelykke, 2014), a mathematical model was established to determine the time of microtubule capture at the immune synapse (Sarkar et al., 2019). Here, microtubules grow and shrink from the MTOC thereby probing their vicinity for a proper anchor (“search”). Once they encounter dynein at the center of the immune synapse they bind to it in an end-on fashion (“capture”). This model incorporates the requirement of dynamic microtubule filaments for MTOC translocation and recapitulates efficient target capture with timescales comparable to experimental data. Search times largely depend on the relative size of the cell, the number of searching microtubules and the distance of the MTOC from the nuclear surface. They become minimal when the immune synapse forms at the nearest or at the farthest sites on the cell surface with respect to the perinuclear MTOC.

### MTOC Translocation in Antigen-Presenting Cells

Microtubule organizing center polarization toward the immune synapse was also reported in antigen-presenting cells such as dendritic cells and B cells (Pulecio et al., 2010; Yuseff et al., 2011). In both cell types, reorientation depends on the small GTPase Cdc42 and its upstream activator DOCK8 (Randall et al., 2011). In dendritic cells, vesicles containing the pro-inflammatory cytokine IL-12 enrich around the MTOC immediately after T cell receptor ligation and become dragged toward the dendritic cell-T cell interface suggesting, that MTOC polarization in dendritic cells enables local priming of T cells (Pulecio et al., 2010). However, ultrastructural studies of T cell-dendritic cell conjugates and time-lapse videomicroscopy of plasmacytoid dendritic cell-T cell interactions did not reveal MTOC positioning close to the plasma membrane indicating that MTOC translocation in dendritic cells is dispensable for efficient T cell activation (Mittelbrunn et al., 2009; Ueda et al., 2011). In B cells, MTOC translocation is accompanied by repositioning of lysosomes, which rapidly move toward the B cell receptor-antigen interface and become locally released, allowing acidification of the B cell synapse and secretion of lysosomal proteases that promote the extraction of immobilized surface antigen. Inhibition of lysosome exocytosis results in compromised intracellular antigen processing and presentation to T cells (Duchez et al., 2011; Yuseff et al., 2011).

**FIGURE 4:**
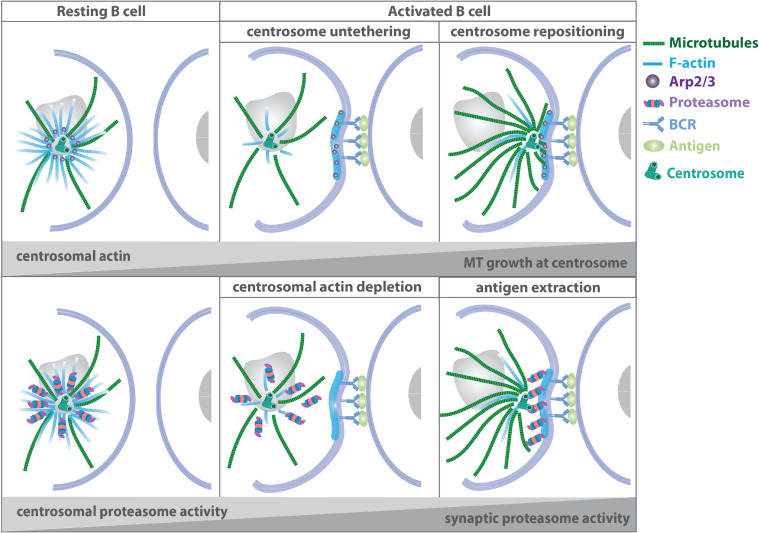
Microtubule-actin crosstalk at the B cell synapse. Upper panel: in resting B cells, Arp2/3-mediated actin nucleation at the centrosome tethers the MTOC to the nucleus. Upon B cell receptor activation, Arp2/3 and F-actin are recruited to the immune synapse thus facilitating centrosome-nucleus separation and repositioning of the MTOC to the cell-cell contact side. Lower panel: proteasomal activity is required for centrosomal actin depletion and subsequent centrosome relocalization to the immune synapse (Obino et al., 2016; Ibañez-Vega et al., 2019).

The mechanisms of MTOC translocation in B cells rely on the interplay of actin and microtubule filaments at the centrosome: in addition to its role as MTOC, the centrosome functions as an organizer of a local network of actin filaments, which can impose physical constraints on the microtubule cytoskeleton, specifically affecting the growth and shape of microtubule filaments as well as the dynamics of the centrosome (Piel et al., 2000; Rodriguez et al., 2003; Huber et al., 2015; Colin et al., 2018; Dogterom and Koenderink, 2019). Centrosomal actin therefore directly regulates the amount and number of nucleating microtubule filaments, by acting as a physical barrier that restricts elongation of nascent microtubules (Farina et al., 2015; Inoue et al., 2019). In resting B lymphocytes, actin related protein 2/3 complex (Arp2/3)-mediated actin nucleation tethers the centrosome to the nucleus thus preventing cell polarization (Figure 4). Upon B cell receptor ligation, clearance of centrosomal Arp2/3 reduces F-actin nucleation at the centrosome and facilitates centrosome-nucleus separation allowing the repositioning of the MTOC to the immune synapse. Arp2/3 and F-actin reduction at the centrosome upon B cell activation results from their recruitment to the immune synapse and depends on the Cortactin homolog hematopoietic lineage cell-specific protein (HS1) (Obino et al., 2016). Moreover, proteasome activity is required for centrosomal actin depletion and subsequent centrosome relocalization to the immune synapse. B cells contain two pools of the 26S proteasome, which modulate actin dynamics at the centrosome and at the immune synapse. Proteasome inhibition results in elevated levels of ubiquitinylated proteins and actin at the centrosome, while less F-actin is found at the synaptic membrane. This suggests that upon B cell activation, centrosomal proteasome activity is required to degrade ubiquitylated proteins involved in actin polymerization thus enabling actin depletion and centrosome polarization toward the immune synapse (Ibañez-Vega et al., 2019).

**FIGURE 5:**
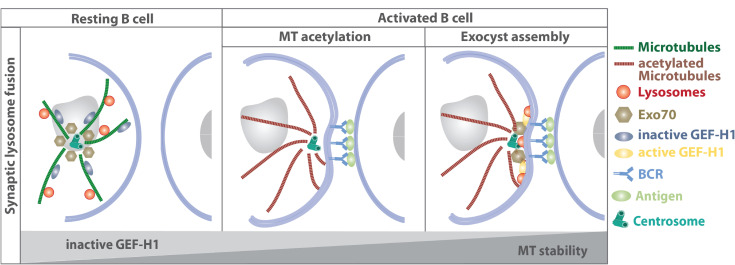
Stabilization of centrosomal microtubules support lysosome fusion at the synaptic membrane by releasing Exo70 and GEF-H1. Concomitantly to the repositioning of the centrosome, lysosomes are recruited to the immune synapse and become locally released, allowing acidification of the B cell synapse and secretion of lysosomal proteases that promote the extraction of immobilized surface antigen. In resting B cells, GEF-H1 is bound to microtubule filaments and kept in its inactive state, while Exo70 is mainly associated to the centrosome. BCR engagement triggers microtubule acetylation, which results in the release of Exo70 and GEF-H1 and subsequent recruitment to the immune synapse. There, GEF-H1 promotes the assembly of the exocyst complex to enable local lysosome fusion at the synaptic membrane (Yuseff et al., 2011; Sáez et al., 2019).

Recently, the mechanistic link between local lysosome fusion at the B cell immune synapse and microtubule dynamics has been addressed (Figure 5): lysosome fusion at the synaptic membrane is supported by stabilization of centrosomal microtubules leading to the redistribution of the exocyst complex to the immune synapse (Sáez et al., 2019). B cell receptor engagement promotes the formation of a stable centrosomal microtubule network, which in turn releases Exo70, a component of the exocyst complex, from the centrosome. Concomitantly, the GTP exchange factor GEF-H1 dissociates from microtubules and together with Exo70 promotes docking and fusion of lysosomes at the immune synapse.

Together, in antigen-presenting cells the direct actin-microtubule crosstalk determines the capacity of the MTOC to repolarize and nucleate microtubules in response to external stimuli and enables local lysosome delivery at the immune synapse.

### Polarized Secretion of Effector Molecules at the Synapse

**FIGURE 6:**
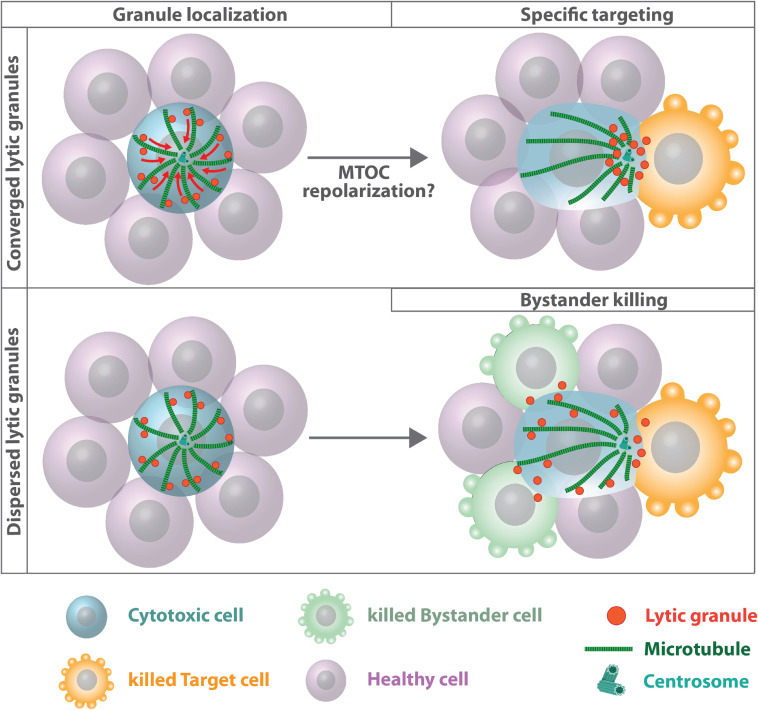
Microtubules and lytic granule convergence. Cytotoxic cells assemble lytic granules around the MTOC, which improves the efficiency of targeted killing (upper panel). Impaired lytic granule convergence results in non-directed degranulation and bystander killing of healthy cells (Hsu et al., 2016). The role of centrosome polarization for efficient cytotoxic T lymphocytes and natural killer cell-mediated killing is still unclear. Under certain experimental conditions, MTOC relocalization toward the immune synapse is dispensable for polarized granule secretion and cytotoxicity (Bertrand et al., 2013; Tamzalit et al., 2020). Under these conditions plus-end directed transport along microtubule filaments might deliver lytic granules to the synapse.

Initially, association of lytic granules with the MTOC and its repositioning toward the immune synapse have been identified as the only prerequisite for targeted secretion while plus-end directed motor transport along microtubule filaments was dispensable for specific killing (Stinchcombe et al., 2006). Lytic granules are delivered directly by the MTOC to an actin-depleted zone within the central SMAC. Yet, granules can also move from the periphery to the synapse using microtubule tracks that are oriented tangentially to the plasma membrane (Poenie et al., 2004). Later studies revealed that granules can take either path depending on the kinetics of downstream T cell receptor signaling (Beal et al., 2009). Lytic granule concentration around the MTOC prior to centrosome reorientation was proposed to increase the capacity of cytotoxic T lymphocytes to elicit a more effective cytolytic response against the next target cell (Stinchcombe and Griffiths, 2007). After leaving a target cell the granules often remain concentrated around the MTOC even when the MTOC repolarizes. This allows cytotoxic T lymphocytes to more rapidly release granules toward the next target cell. A causal link between lytic granule convergence and improved efficiency of target cell lysis was established by Hsu and colleagues demonstrating that concentration of lytic vesicles around the MTOC prevents bystander killing of healthy cells (Hsu et al., 2016; Figure 6). Granule convergence toward the MTOC was identified to occur rapidly (Wiedemann et al., 2006). Vesicles move toward microtubule minus-ends in a dynein-dependent manner but independently of microtubule and F-actin reorganization and commitment to cytotoxicity (Mentlik et al., 2010; Ritter et al., 2015). Dynein-mediated minus-end-directed movement of lytic granules is dependent on Src family kinase activity as well as downstream LFA-1 signaling (James et al., 2013; Zhang M. et al., 2014; Kabanova et al., 2018) and the adaptor Hook-related protein 3 (HkRP3) which links lytic granules to the dynein motor complex possibly via DOCK8 (Ham et al., 2015). More recently, vasodialator-stimulated phosphoprotein (VASP), an actin regulatory protein, was identified to regulate lytic granule convergence in natural killer cells. VASP depletion does not interfere with synapse formation and MTOC reorientation but is essential for efficient lytic granule concentration at the MTOC (Wilton and Billadeau, 2018). Once the MTOC and its associated vesicles polarized toward the plasma membrane, a complex between kinesin-1, Slp3, and Rab27 mediates plus-end directed transport of lytic granules along microtubule filaments and docking to the plasma membrane (Kurowska et al., 2012). Rab27a is also known to interact with Munc13-4 and is important for the final steps of lytic granule exocytosis (Ménager et al., 2007). Cells deficient in removing acetylation sites of α-tubulin due to depletion of histone deacetylase six fail to transport lytic granules to the target cell implying that microtubule dynamics and stability play an important role for terminal vesicle transport (Nunez-Andrade et al., 2016).

Whether centrosome polarization is a prerequisite for efficient cytotoxic T lymphocytes and natural killer cell-mediated killing is still a matter of debate. While in several studies impaired or delayed MTOC translocation as well as abrogation of transient centrosome docking with the plasma membrane is associated with reduced cytotoxicity (Quann et al., 2009; Tsun et al., 2011; Stinchcombe et al., 2015), other studies suggest efficient target cell killing prior to or in the absence of MTOC reorientation (Butler and Cooper, 2009; Bertrand et al., 2013). Recently, genetic depletion of centrioles was shown to have no measurable effect on lytic granule polarization, directionality of secretion and specificity of target killing, arguing for a centriole-independent way of lytic granule convergence and target cell specificity (Tamzalit et al., 2020). Nevertheless, centriole-deficient cytotoxic T lymphocytes exhibit reduced cytotoxic potential due to profound defects in lytic granule biogenesis and synaptic F-actin dynamics, resulting in reduced F-actin clearance at the center of the synapse and decreased synaptic force exertion. Centriole-depleted cytotoxic T lymphocytes still assemble components of the pericentriolar material into acentriolar MTOCs, which retain their ability to nucleate microtubule filaments, leading to a perturbed architecture of microtubule arrays in centriole-depleted cells. Depletion of microtubule filaments with nocodazole blocks centrosome reorientation and accumulation of lytic granules at the immune synapse but does not affect the directionality of granule release and bystander killing indicating that the microtubule cytoskeleton is important for facilitating transport of lytic granules to the immune synapse, but centrosome polarization *per se* is not essential for the spatial specificity of fusion events (Tamzalit et al., 2020).

### Concluding Remarks and Future Perspectives

Here we have summarized the multiple faces of microtubule involvement during immunological processes ranging from leukocyte migration to immune synapse formation and vesicle polarization. We have learned from recent studies that leukocyte microtubules are essential for the induction of tissue immunity by maintaining cell coherence of migrating leukocytes and regulating polarized secretion of granules and lysosomes. As such, microtubule arrays deserve precise attention and exact description in future studies of immunological processes.

Migrating dendritic cells exhibit differences in the dynamics of front- versus back-directed microtubule filaments. How this polarization is achieved and what are the key regulators of microtubule dynamics in migrating leukocytes still remain open questions. Advanced light sheet microscopy in combination with recently developed photo-switchable compounds and optogenetic constructs that target microtubule stability and dynamics are potent tools to investigate subcellular microtubule dynamics with high spatiotemporal resolution allowing to understand the functional principles of microtubule dynamics in tissue navigating cells (Borowiak et al., 2015; Müller-Deku et al., 2020; Van Geel et al., 2020). Similarly, the regulatory mechanisms of microtubule dynamics during immune synapse formation and polarized vesicle release are poorly understood. Plus-end binding proteins are essential regulators of polarized microtubule stability and density where EB1-dependent microtubule remodeling is crucial for vesicle trafficking and sustained T cell activation (Martin-Cofreces et al., 2012). Yet, how EBs are regulated at the plus-ends of microtubules and which factors regulate microtubule density during immune synapse formation is currently ill defined.

Adaptive immunity is to a large extent influenced by mechanical forces. Microtubules accumulate a great diversity of PTMs, which can alter microtubule dynamics and thus control the mechanical properties and function of microtubule filaments. Acetylation of α-tubulin at the luminal surface of microtubules affects filament stability and is associated with long lived microtubule subpopulations (Howes et al., 2014; Eshun-Wilson et al., 2019). Functionally, tubulin acetylation is required to protect microtubules from mechanical breakage indicating that acetylation increases mechanical resilience of microtubule filaments (Portran et al., 2017; Xu et al., 2017). Moreover, acetylation facilitates bundling of microtubules, which in turn enhances kinesin activity along microtubule tracks (Balabanian et al., 2017). In this regard, increased motor activities due to tubulin acetylation could promote sliding forces, which allow MTOC repositioning toward the immune synapse (Serrador et al., 2004).

During dendritic cell migration acetylation occurs predominantly on front directed microtubules (Serrador et al., 2004; Kopf et al., 2020). In particular microtubules reaching the base of the lamellipodium experience high forces from actin-based retrograde flow, which in mesenchymal cells influences microtubule dynamic instability and turnover (Waterman-Storer and Salmon, 1997). Stabilization of microtubules by acetylation could thus contribute to resist these forces and allow microtubules to grow into explorative protrusions.

In the future, it will be interesting to employ life cell sensors to analyze the role of PTMs in a detailed spatiotemporal manner during processes such as leukocyte migration and immune synapse formation. The tools are currently limited; however, recent advances now enable monitoring of tyrosinated microtubules in living cells (Kesarwani et al., 2020).

Moreover, T cell activation and effector function is a mechanosensitive process, in which force transduction across the T cell receptor complex generates significant traction stresses at the cell-cell interface (reviewed by Blumenthal and Burkhardt, 2020). These stresses are essential for enabling antigen recognition and T cell activation. Dynamic microtubules have recently been described as critical determinants of force generation during T cell activation by locally modulating actomyosin activation (Hui and Upadhyaya, 2017). However, whether and how a dynamic microtubule cytoskeleton also contributes to the force generating processes of antigen-presenting cells, and whether this is important for mounting an accurate immune response, is currently poorly understood. Therefore it will be crucial to decipher how mechanical coupling between the actomyosin system and the microtubule cytoskeleton affects immune synapse formation and activation of T cells.

Another interesting feature of leukocytes that has received limited attention during the past years is the centrosome-nucleus connection. It is well established that stationary or slow-moving cells such as neurons or cells of mesenchymal origin exhibit a stable front-back polarization (Meiring et al., 2020). However, highly motile leukocytes violate this paradigm and exhibit dynamic polarity configurations where the centrosome can be found in various positions with respect to the nucleus (Chiplonkar et al., 1992). Even more extreme is the flexible centrosomal-nuclear coupling distance of leukocytes. This is exemplified in migrating dendritic cells that reside in bifurcating channels, where in about 20% of the cases the MTOC uncouples transiently from the nucleus during path selection (Renkawitz et al., 2019). However, after path selection the centrosomal-nuclear distance decreases again. This behavior is to some extent similar to nucleokinesis in neurons in which the centrosome first changes its position into a leading process which is followed by nuclear movement toward the centrosome (Minegishi and Inagaki, 2020). The linker of nucleoskeleton and cytoskeleton (LINC) together with other molecular players such as Lissencephaly 1 (LIS1), myosin II activity and substrate topology contribute to centrosomal-nuclear axis orientation in many cell types (Luxton and Gundersen, 2011; Chang et al., 2015; Zhu et al., 2017) but the precise molecular mechanism of centrosome repositioning and centrosome-nucleus coupling remain largely unexplored in leukocytes.

The immune synapse is a well-known site of polarized membrane transport and localized vesicle release. While this process is fairly well understood in T cells and cytotoxic cells, it is less clear how polarized secretion is regulated in antigen-presenting cells. MTOC relocalization in dendritic cells seems to be dispensable for the specific delivery of cytokines and T cell activation. As dendritic cells are able to interact and activate several T cells simultaneously (Miller et al., 2004) it seems favorable to nucleate microtubule filaments from the centrally located centrosome instead of a polarized MTOC. Computer simulations based on a general random velocity model predict efficient cargo delivery along microtubule filaments to specific target areas on the plasma membrane without MTOC translocation (Hafner and Rieger, 2016). Based on these simulations it is tempting to speculate that MTOC translocation constitutes an efficient strategy in favor of single cell-cell contacts while a centrally located MTOC promotes the formation of several active synapses. Future experiments yet have to clarify the role of MTOC polarization in antigen-presenting cells such as dendritic cells.

In contrast to mesenchymal cells, many of the core cell biological characteristics of the leukocyte microtubule cytoskeleton are currently poorly understood. Given that chemokines and other external factors, such as the mechanochemical composition of the microenvironment, act upstream of cytoskeletal regulation (Jin et al., 2019) it will not be surprising to discover new aspects and molecular mechanisms of microtubule regulatory functions in leukocytes. Since a large number of essential processes converge upon the microtubule cytoskeleton, understanding how microtubules support leukocyte functionality is critical to understanding the physiology and pathophysiology of immunological processes and will open up new strategies for targeted interventions.

## Author Contributions

Both authors contributed to the conceptual development of the work, literature research, and writing of the manuscript. AK composed the figures.

## Conflict of Interest

The authors declare that the research was conducted in the absence of any commercial or financial relationships that could be construed as a potential conflict of interest.
